# Unveiling the Role of Nonionic Surfactants in Enhancing Cefotaxime Drug Solubility: A UV-Visible Spectroscopic Investigation in Single and Mixed Micellar Formulations

**DOI:** 10.3390/ph16121663

**Published:** 2023-11-29

**Authors:** Aysha Arshad Rana, Amnah Yusaf, Salma Shahid, Muhammad Usman, Matloob Ahmad, Sana Aslam, Sami A. Al-Hussain, Magdi E. A. Zaki

**Affiliations:** 1 Department of Chemistry, Government College Women University, Faisalabad 38000, Pakistan; 2 Department of Biochemistry, Government College Women University, Faisalabad 38000, Pakistan; 3 Department of Chemistry, Government College University, Faisalabad 38000, Pakistan; 4 Department of Chemistry, College of Science, Imam Mohammad Ibn Saud Islamic University (IMSIU), Riyadh 11623, Saudi Arabia

**Keywords:** cefotaxime, solubilization, nonionic surfactants, mixed micelle, drug delivery, encapsulation efficiency, partition coefficient

## Abstract

This study reports the interfacial phenomenon of cefotaxime in combination with nonionic surfactants, Triton X-100 (TX-100) and Tween-80 (TW-80), and their mixed micellar formulations. Cefotaxime was enclosed in a micellar system to improve its solubility and effectiveness. TX-100 and TW-80 were used in an amphiphilic self-assembly process to create the micellar formulation. The effect of the addition of TX-100, a nonionic surfactant, on the ability of TW-80 to solubilize the drug was examined. The values of the critical micelle concentration (CMC) were determined via UV-Visible spectroscopy. Gibbs free energies (ΔG_p_ and ΔG_b_), the partition coefficient (K_x_), and the binding constant (K_b_) were also computed. In a single micellar system, the partition coefficient (K_x_) was found to be 33.78 × 10^6^ and 2.78 × 10^6^ in the presence of TX-100 and TW-80, respectively. In a mixed micellar system, the value of the partition coefficient for the CEF/TW-80 system is maximum (5.48 × 10^6^) in the presence of 0.0019 mM of TX-100, which shows that TX-100 significantly enhances the solubilizing power of micelles. It has been demonstrated that these surfactants are effective in enhancing the solubility and bioavailability of therapeutic compounds. This study elaborates on the physicochemical characteristics and solubilization of reactive drugs in single and mixed micellar media. This investigation, conducted in the presence of surfactants, shows a large contribution to the binding process via both hydrogen bonding and hydrophobic interactions.

## 1. Introduction

There is a continuous trend in the rising number of new medications with more complicated molecular structures, and subsequently, the problem of worse solubility is continuously rising. According to previous studies, up to 90% of the drugs being investigated are not soluble in water [1]. Drugs which are poorly water-soluble require the use of formulation strategies to increase their bioavailability [2]. Less water-soluble drugs might even result in unreliable and inconsistent consumption, while excessive drug doses can cause different side effects, such as gastrointestinal issues, poisoning, cardiovascular effects, central nervous system effects, and respiratory issues [3]. Improvements in the water solubility of pharmaceuticals and effectiveness of encapsulation are thus desired to enhance drug absorption, improve bioavailability, and decrease the number of required therapeutic doses [4].

Drug delivery systems are described as formulations that are designed to move a medicine to the intended site of action inside the body more efficiently. A suitable carrier that shields the drug from quick degradation and increases its concentration within the target tissues is the fundamental component in drug delivery systems [5]. Amphiphilic compounds known as surfactants, which can lower the surface tension amongst two immiscible phases, are prospective drug transporters [6]. The molecules of these substances have both a hydrophilic and a hydrophobic component in the same moiety [7]. Because of their structural characteristics, surfactants can function as emulsifiers, detergents, foaming agents, floating agents, solubilizers, drug-delivery agents, etc., both in daily life and in industrial settings [8]. Surfactants can increase the solubility of drugs because they can create colloidal-sized microemulsions (micelles) in specific liquids [9]. Because surfactants have amphiphilic nature, their molecules self-assemble into micelles at concentrations above the critical micelle concentration (CMC) [10]. Due to their ability to form micelles, amphiphilic surfactants have attracted a lot of attention recently for use in the delivery of drugs. The former studies include the solubilization of drugs in single and mixed micellar systems of surfactants. Single micellar systems refer to formulations consisting solely of one type of surfactant, such as TX-100 (TX-100) or Tween-80 (TW-80), providing a homogenous micellar environment [11]. Conversely, mixed micelles involve the combination of two or more surfactants, such as TW-80 and TX-100, creating a heterogeneous micellar system [12]. In comparison to their individual constituent surfactants or drugs, the mixture of drug–surfactant exhibited a synergistic effect and promised more desirable properties. A synergistic relationship exists between the increase in interface activity of the mixed micellar system and decreased critical micelle concentration (CMC) [13].

Micellar systems provide particular advantages that traditional systems, such as liposomes, may not fully replicate. Micellar systems have an advantage over liposomal systems [14] in that they can encapsulate a wider range of hydrophobic substances and modify their composition more easily, perhaps providing improved drugs solubility [15]. Although nanocrystals improve drugs’ dissolving rates, micellar systems stand out due to their targeted drug delivery capabilities [16]. Micelles may change their surface features for selective targeting, a feature uncommon in nanocrystal formulations, enabling for precision in medication delivery to target tissues or cells. The therapeutic efficacy of any particular drug is influenced by its ability to reach the defined target organs, tissues, or cells. The surfactant enables it to behave as a carrier, enhancing the delivery of the drug to a target site [17]. As a way to enhance the therapeutic outcomes of powerful drugs, surfactants have already been widely used in pharmaceutical science as excipients for conventional pharmaceutical formulations [18]. Amphipathic compounds named surfactants have non-polar and polar substituents in their heads and tails, respectively.

They can be categorized as anionic, cationic, amphoteric, or nonionic surfactants due to the difference in charges that exists on the head groups [19]. The existence of a dual-characteristic component facilitates the self-aggregation of monomers into micelles at a certain concentration within the aqueous environment, termed the critical micelle concentration (CMC) [20]. Micellar solubilization is an excellent method for increasing drug solubility under physiological conditions in the body [21]. The drug can dissolve in a surfactant solution at CMC concentration, and the dissolution rate rises further with higher surfactant concentrations [22].

One significant class of amphiphiles is nonionic amphiphiles with ethylene oxide sequences (hydrophilic group). The solubilization power of nonionic surfactants is frequently greater than that of anionic and cationic surfactants. Nonionic surfactants have a significant role in pharmacology because of their low protein binding and high solubility at low CMC scales. They enhance the performance and availability of the drug as well. Additionally, their micellar system is not affected by significant dilution in the blood [23]. Nonionic amphiphiles are widely employed in both commercially available pharmaceutical formulations and other industries due to their unique characteristics.

Nonionic amphiphiles also produce thermodynamically stable nanoscale assemblies known as “micelles”, although at lower concentrations than other amphiphiles. Micelles are one of the many delivery systems that have been created up to this point. They tend to be homogeneous and thermodynamically stable when immersed in water and are created when surfactants self-assemble above a critical micellar concentration (CMC). Nonionic amphiphiles are used in commercial and pharmaceutical formulations because of their intriguing features. Micelles, which have a diameter ranging from 10 to 100 nm, are thought to be a simple and effective environment for dissolution of insoluble drugs because of their distinctive amphiphilic nature, with a hydrophilic crust and a hydrophobic core [24]. The effectiveness of amphiphilic drugs increases, with fewer adverse effects, when combined with a carrier (surfactants) [25].

Nonionic amphiphiles also produce thermodynamically stable nanoscale assemblies known as “micelles”, although at lower concentrations than other amphiphiles. Micelles are one of the many delivery systems that have been created up to this point. They tend to be homogeneous and thermodynamically stable when immersed in water and are created when surfactants self-assemble above a critical micellar concentration (CMC). Nonionic amphiphiles are used in commercial and pharmaceutical formulations because of their intriguing features. Micelles, which have a diameter ranging from 10 to 100 nm, are thought to be a simple and effective environment for dissolution of insoluble drugs because of their distinctive amphiphilic nature, with a hydrophilic crust and a hydrophobic core [24]. The effectiveness of amphiphilic drugs increases, with fewer adverse effects, when combined with a carrier (surfactants) [25].

Micellar systems employ a solubilization technique by encapsulating the Active Pharmaceutical Ingredient (API), i.e., the drug, within their hydrophobic cores, typically formed by surfactant molecules. This encapsulation process enables hydrophobic drugs, which have poor solubility in water, to be incorporated and dispersed in the aqueous environment of the micellar solution [26]. The hydrophobic tails of surfactant molecules surround the drug, shielding it from the aqueous medium. The hydrophobic feature of the micellar core is employed in order to dissolve drugs in an aqueous solution, whereas the existence of hydrated counter ions stabilizes the outer layer of the micelles. Therefore, interactions between the drug and the surfactant that are both electrostatic and hydrophobic determine where the drug is located in the micellar system [27]. A safe path for the drug to make its way to the intended site is provided by micellar systems, which lower the likelihood of drug degradation and increase its bioavailability [28]. The study of surfactant agglomeration and its possible applications is an important issue, particularly in connection with studies on the impact of organized assemblies during different chemical transformations [29]. Pharmaceutical formulations are said to use amphiphilic drugs as part of the drug delivery mechanism to enhance the affinity and solubilization capabilities of weakly water-soluble drugs [13]. It was discovered that the chemical makeup of drugs and surfactants affects their ability to form self-nanoemulsions [30]. Cefotaxime is a potent antibiotic with a well-known structure. Belonging to the third generation of cephalosporins, this molecule has a variety of active sites and is employed as an antibiotic. Cefotaxime (CEF) has proven effective in treating a wide range of bacterial infections and has played a significant role in limiting bacterial growth, particularly in preventing infections in different surgical settings [31].

The objective of the current research is to use UV-Visible absorption measurements to examine the pharmacological potential of micellar solubilization of cefotaxime in nonionic surfactants in order to catalyze drug delivery systems (in both single and mixed micellar systems). We looked at how the drug affected the CMC of TX-100 and Tween 80, which are used as carriers to deliver the drug into the body. Complex measurements of the CEF-TX-100 and CEF-TW-80 were also used to assess a number of related factors.

## 2. Results and Discussion

### 2.1. Simple UV-Visible Absorption Spectra

**Figure 1:**
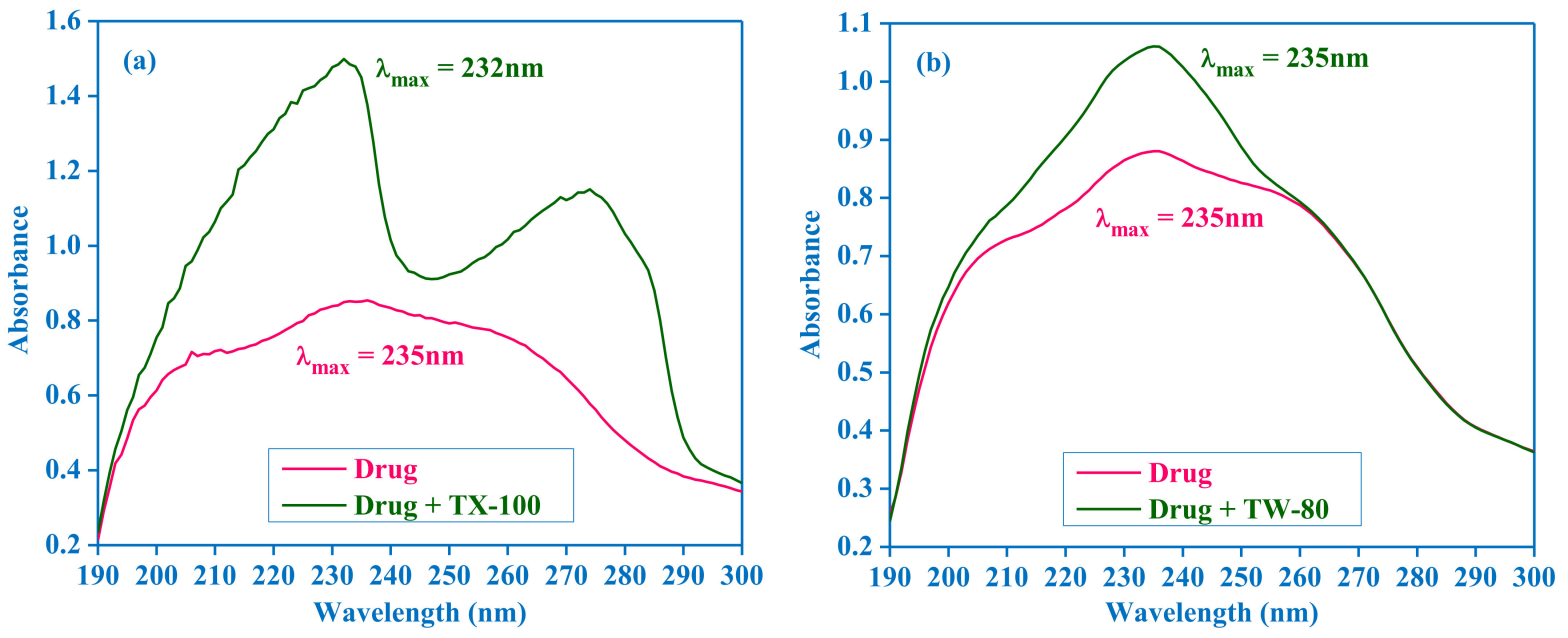
Plot of UV-Visible absorption spectra of CEF in aqueous and micellar media of TX-100 (**a**) and TW-80 (**b**).

The UV-Visible spectra of pure CEF, as well as CEF in the TX-100 and TW-80 micelles, are displayed in Figure 1.

First, a graph between absorbance and wavelength was plotted to identify the λ_max_. Then, surfactant absorbance values at λ_max_ were noted. In the presence of TX-100 and TW-80, the drug exhibited a hyperchromic shift. The presence of nonionic surfactants in the aqueous solution of the drug caused a slightly high redshift in the absorption band position. A higher concentration of surfactant, above CMC, leads to the formation of a large number of micelles. These micelles have the ability to bind and contain drug molecules inside their closed structures. The aforementioned phenomenon increases the intensity of absorption [32,33]. The λ_max_ of CEF was observed at 235 nm. The lack of a shift in the max after TX-100/TW-80 administration to the drug solution can be explained by the nonionic surfactant concentration being significantly above CMC. Surfactant molecules form micelles in the solution when their hydrophobic tails combine, but their hydrophilic heads remain exposed to the solvent at concentrations above the CMC. Cefotaxime probably does not have significant interactions with the hydrophilic heads of surfactants. These non-specific interactions do not significantly change the chromophores or electrical characteristics that cause cefotaxime to absorb light. The maximum wavelength is therefore unaffected. Although these micelles can dissolve hydrophobic materials, they often have little to no effect on the electronic transitions of other molecules in the solution [34]. The concentration of surfactant improved the UV-Visible absorbance of CEF, as shown in Figure 1. This figure shows the extensive integration of drug molecules into the micellar system of TX-100 and TW-80. The increase in drug absorption in the pre-micellar region demonstrates the improved bioavailability of drug molecules [19].

**Figure 2:**
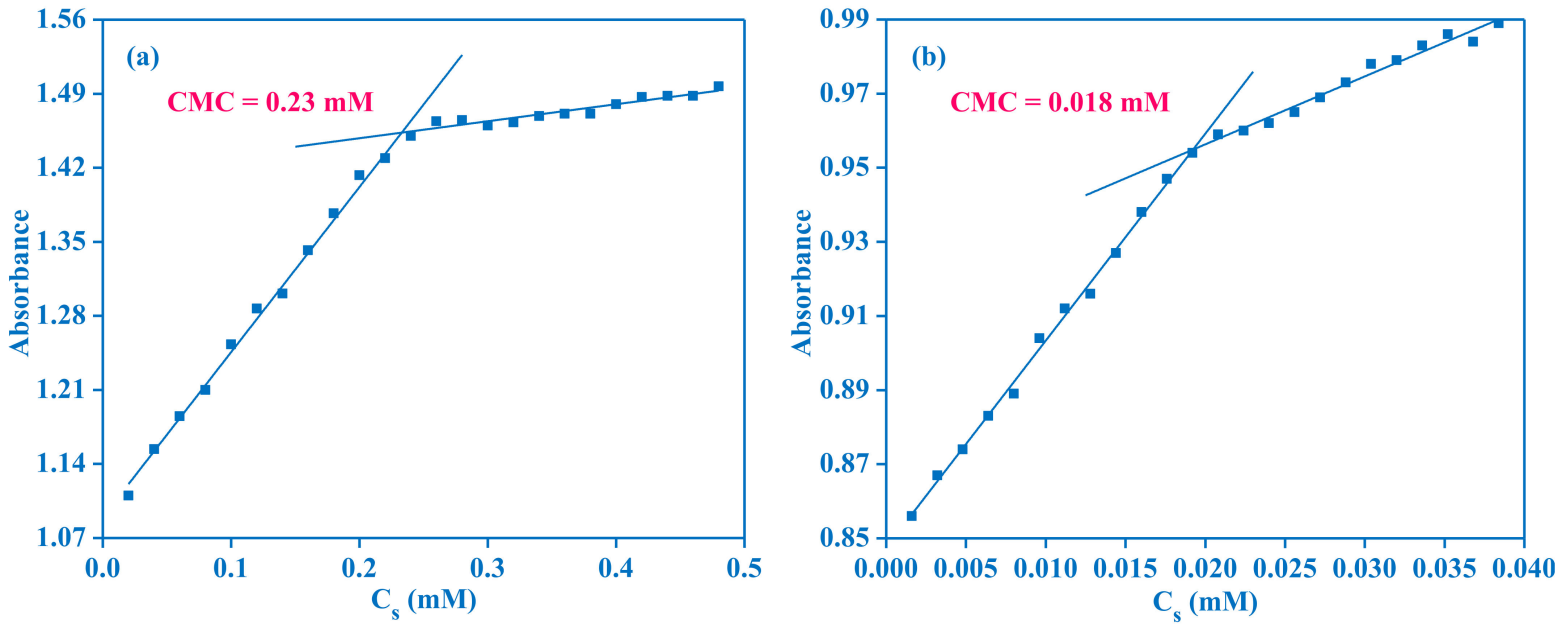
Plot of simple absorbance of CEF against concentration of (**a**) TX-100 and (**b**) TW-80.

Drug absorbance increases rapidly in the post-micellar region up until CMC and then gradually drops when the drug is fully incorporated into the micelle, as shown in Figure 2. However, because more drug molecules are absorbed by freshly formed micelles, absorbance might occasionally increase slowly even after CMC [35]. The typical CMC values of TX-100 and TW-80 in water are 0.2 mM [36,37] and 0.015 mM [38], respectively. The CMC value is increased to 0.24 and 0.018, respectively, after CEF is added to the aqueous solution of TX-100 and TW-80. This is because of the structure-breaking effect of CEF, which destroys water structure and increases entropy, rendering the micellization entropically unfavorable and increasing CMC [39].

### 2.2. Differential UV-Visible Absorption Spectra

#### 2.2.1. Single Micellar System

**Figure 3:**
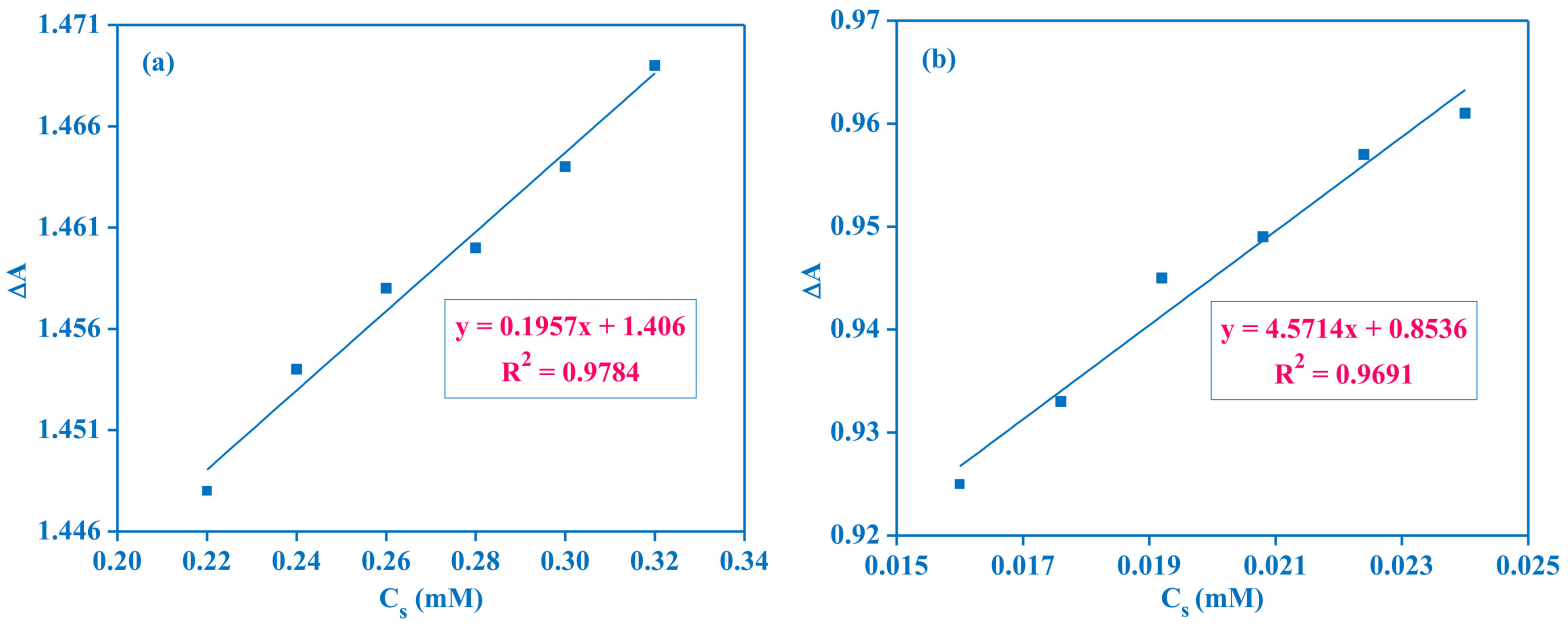
Plot of differential absorbance of CEF as a function of concentration of (**a**) TX-100 (**b**) TW-80.

The relationship between differential absorbance and surfactant concentration is clearly depicted in Figure 3. Here, an increase in the differential absorbance was observed. A gradual increase in the ΔA values with an increase in surfactant concentration indicates that drug molecules are still being incorporated from the aqueous associate with the micelles formed by TX-100/TW-80 systems [40,41]. The data obtained for the differential study of TX-100/TW-80 systems is tabulated in Table 1 and Table 2, respectively, and calculations for partitioning and binding constants were performed using Equations (1) and (5).

**Figure 4:**
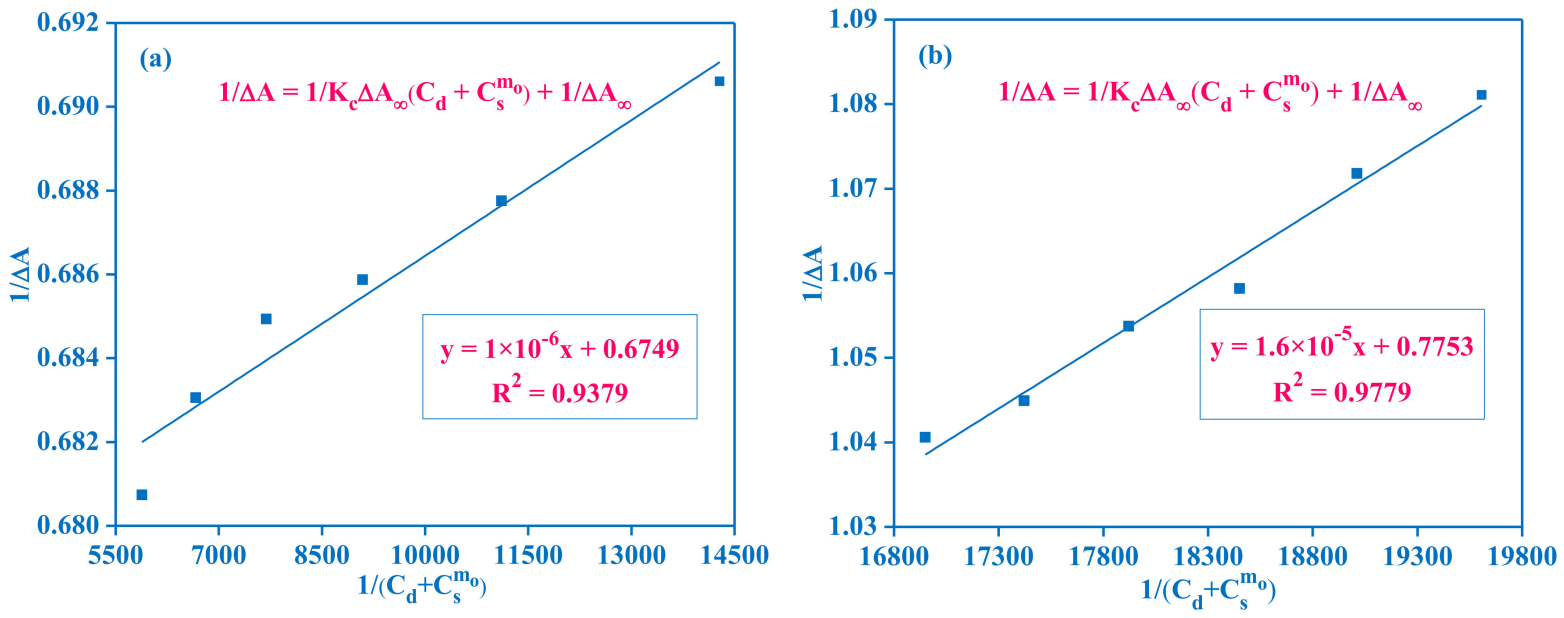
Kawamura plot between 1/ΔA and 1/(C_s_ + $C_{s}^{m_{o}}$) for calculation of K_x_ for (**a**) CEF/TX-100 system (**b**) CEF/TW-80 system.

**Figure 5:**
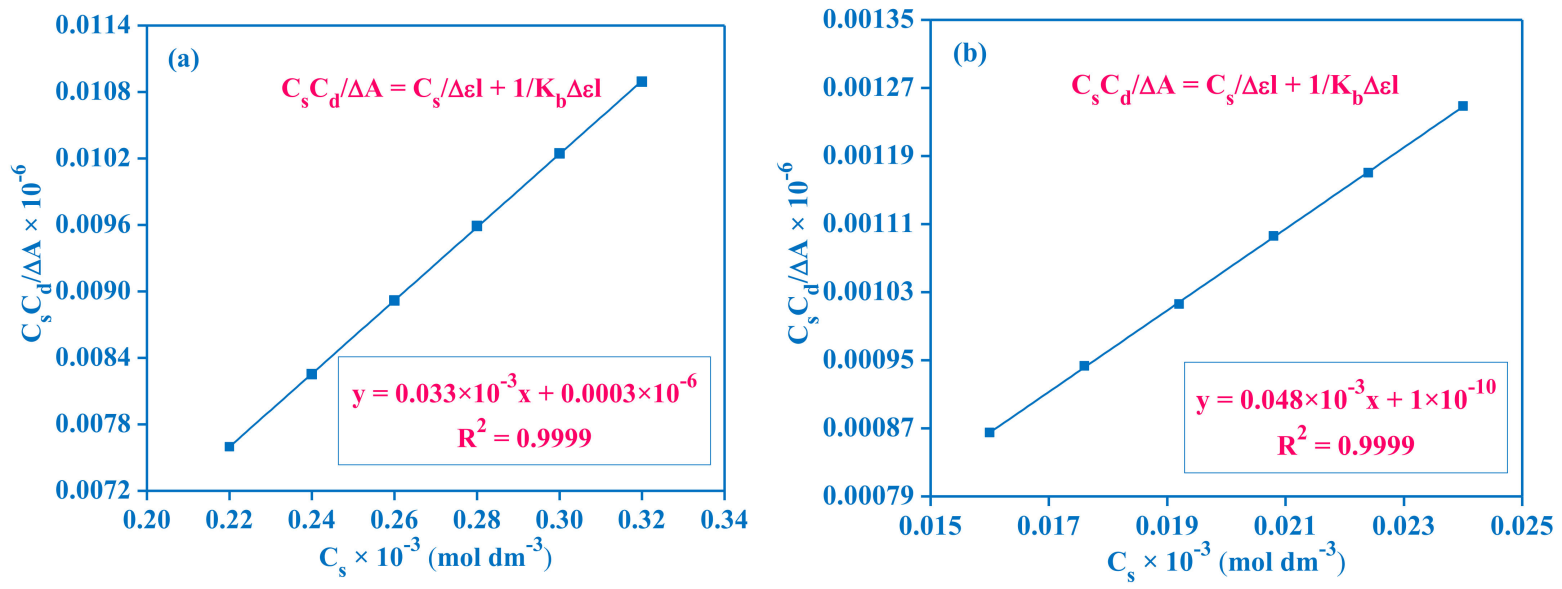
Plot for calculating binding constant (K_b_) for (**a**) CEF/TX-100 system and (**b**) CEF/TW-80 system.

The differential absorbance data, expressed in terms of K_x_ and K_b_, can be used to calculate the degree of solubilization. The binding constant equation and the Kawamura equations were used to calculate these parameters. Furthermore, the potential of binding and partitioning mechanisms was assessed based on their free energies. The Gibbs partitioning and binding energy have negative values, confirming the spontaneity of the solubilization process [42]. Figure 4 and Figure 5 show plots for K_x_ and K_b_ of the TX-100/TW-80 + CEF micellar system, whereas Table 3 displays the values of the partitioning coefficient (K_x_), binding coefficient (K_b_), Gibbs partitioning energy ($\Delta G_{p}$) and Gibbs binding energy ($\Delta G_{b}$).

When the partition coefficients for the dispersion of cefotaxime molecules between water and micellar phases are compared, the value of K_x_ (33.78 ×  10^6^) for TX-100 is greater than that for TW-80 (2.78 ×  10^6^). TX-100 has a higher micellar partition coefficient than TW-80 due to its higher aggregation number (approximately 142) [43,44,45], compared to TW-80 (approximately 60) [46,47]. This difference in aggregation number is responsible for larger micellar size in Triton X-100, allowing for more drug monomers per micelle. Furthermore, the hydrophilic-lipophilic balance (HLB) value for TX-100 is approximately 13.4 compared to Tween 80, which has an HLB value of around 15.0. This higher HLB value suggests overall a slightly greater hydrophilicity for Tween 80 [48]. Cefotaxime, which is hydrophobic in nature, exhibits better partitioning with lower HLB values, i.e., Triton X-100. In contrast, when the binding coefficients for the micellar phases of TX-100 and TW-80 are compared, the higher K_b_ value for TW-80 (4.83 ×  10^5^) compared to TX-100 (0.98 ×  10^5^) is due to the bigger nonpolar tail in Tween-80. This feature generates a stronger complex formation, showing a link between binding and hydrophobicity. It has also been discovered that the interior of the micelles of surfactants with very low CMC is more hydrophobic than the interior of the micelles of identical surfactants with greater CMC [49,50,51]. The values of the Gibbs energy of partition, ΔG_p_, are −43.46 kJ/mol for the CEF/TX-100 system and −37.20 kJ/mol for the CEF/TW-80 system, whereas the Gibbs energy of binding, ΔG_b_, is −28.47 kJ/mol for the CEF/TX-100 system and −32.43 kJ/mol for the CEF/TW-80 system.

#### 2.2.2. Mixed Micellar System

When micelles spontaneously form, two variables come into play. The non-polar part of the molecule is first isolated from water and encased inside the structure as a result of the hydrophobic effect. The second factor determining how tightly the molecules can be packed is the interactions between the head groups [52]. Mixed micelles have more surfactant. In surfactant formulations that exhibit synergism when combined, the aggregation number rises. In surfactant systems with strong interactions, the aggregation number is greater (as seen in mixed systems of TX-100 and TW-80), indicating micellar development in comparison to single surfactant micelles [53]. The rate of solubilization within the micellar core increases with the mixed micelle’s aggregation number, whereas it decreases in the palisade layer due to the strong interactions among the surfactant head groups. A beneficial synergism is expected when a larger amount of the solubilizate resides in the micellar core. On the other hand, if the solubilization of the palisade layer exceeds, the mixed micellar solubilization capability is lower than that of pure micelles.

**Figure 6:**
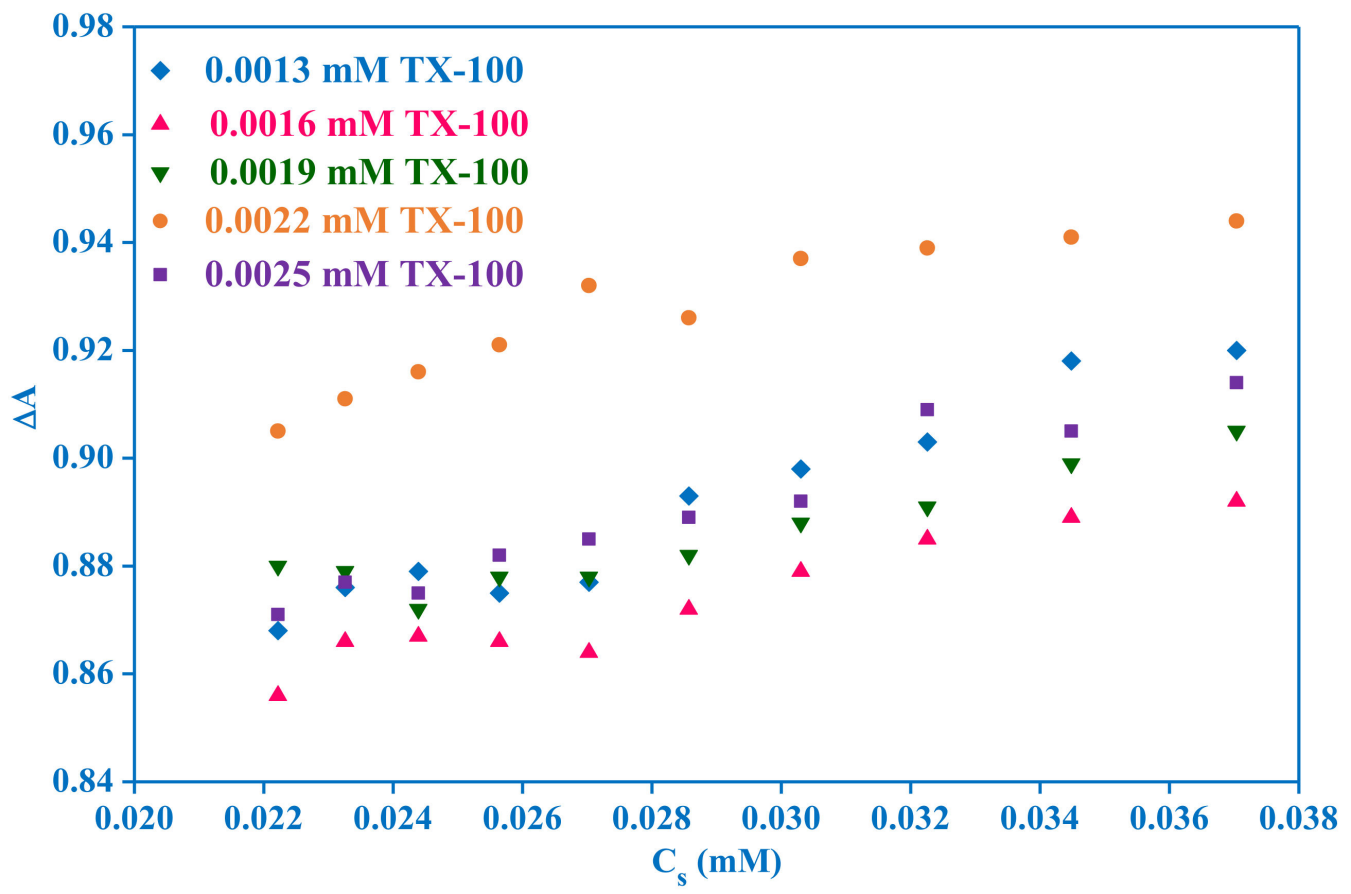
Plot of the differential absorbance of CEF in a mixed micellar system with concentrations of TX-100 and TW-80.

**Figure 7:**
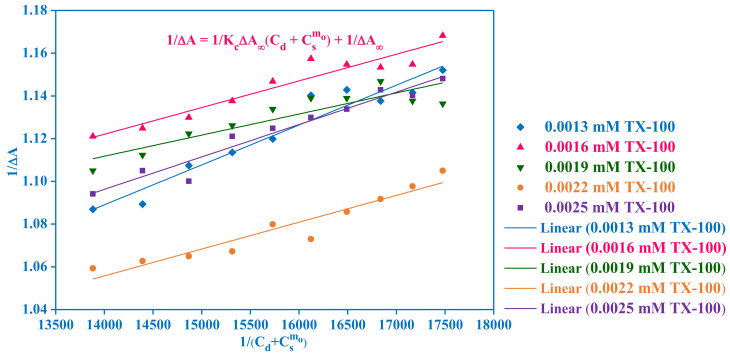
Kawamura plot between 1/ΔA and 1/(C_s_ + $C_{s}^{m_{o}}$) for the calculation of K_x_ in a mixed micellar system.

**Figure 8:**
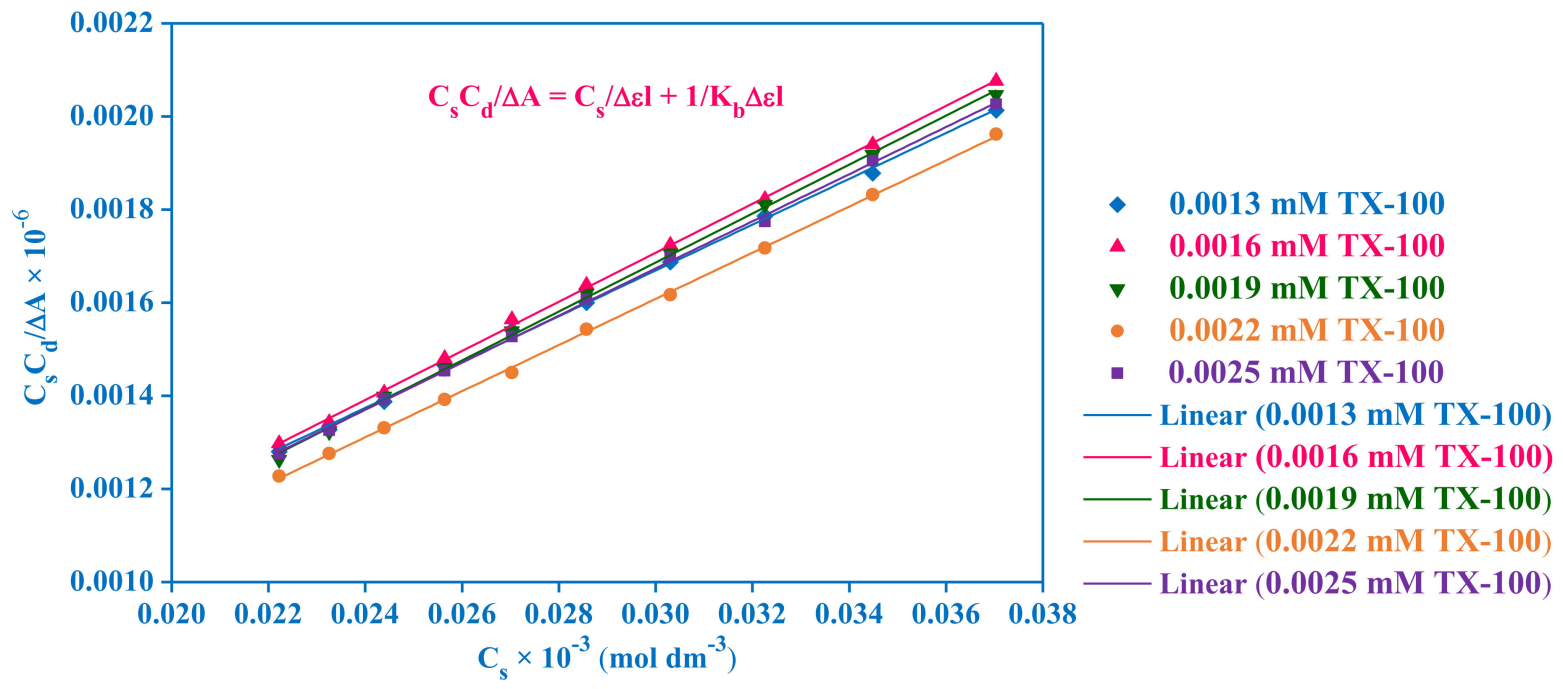
Plot for the calculation of the binding constant in a mixed micellar system.

In the current study, the solubilization of CEF was executed in the micellar system of TW-80 in the presence of 0.0013, 0.0016, 0.0019, 0.0022, and 0.0025 mM of TX-100 as shown in Figure 6. Figure 7 and Figure 8 show plots for K_x_ and K_b_, whereas Table 4 indicates that the value of the binding constant (K_b_) increases with an increase in the concentration of TX-100 because of stronger interactions of CEF with the mixed micellar solution of TW-80. The steric self-repulsion due to a larger headgroup size of TW-80 (20 oxyethylene groups) becomes less potent after being combined with TX-100 and the steric self-repulsion is replaced by dipole-dipole interactions between the hydrophilic head groups of the nonionic surfactants [54]. Despite this, the increasing pattern in the solubilizing strength of mixed micellar systems with concentrations of TX-100 is only apparent up to a certain point because, after an initial increase, the values of K_x_ and K_b_ decrease, leading to a drop in partitioning. Beyond a certain threshold concentration of TX-100, the micellar system reaches saturation in terms of solubilizing capacity. This saturation limits the incorporation or solubilization of the drug within the micelles despite increased TX-100 concentrations, leading to a decline in partitioning. It is also believed that at a greater concentration of TX-100, the packing of TX-100 and TW-80 in the mixed micelle reduces the space available for dye partitioning [55]. The value of the partition coefficient for the CEF/TW-80 system is maximum (5.48 × 10^6^) in the presence of 0.0019 mM of TX-100, demonstrating that TX-100 considerably increases the ability of micelles to dissolve.

### 2.3. Study of Interaction Mechanisms

#### 2.3.1. Binding Nature of Cefotaxime in a Single Micellar System with TX-100 and TW-80

**Scheme 1:**
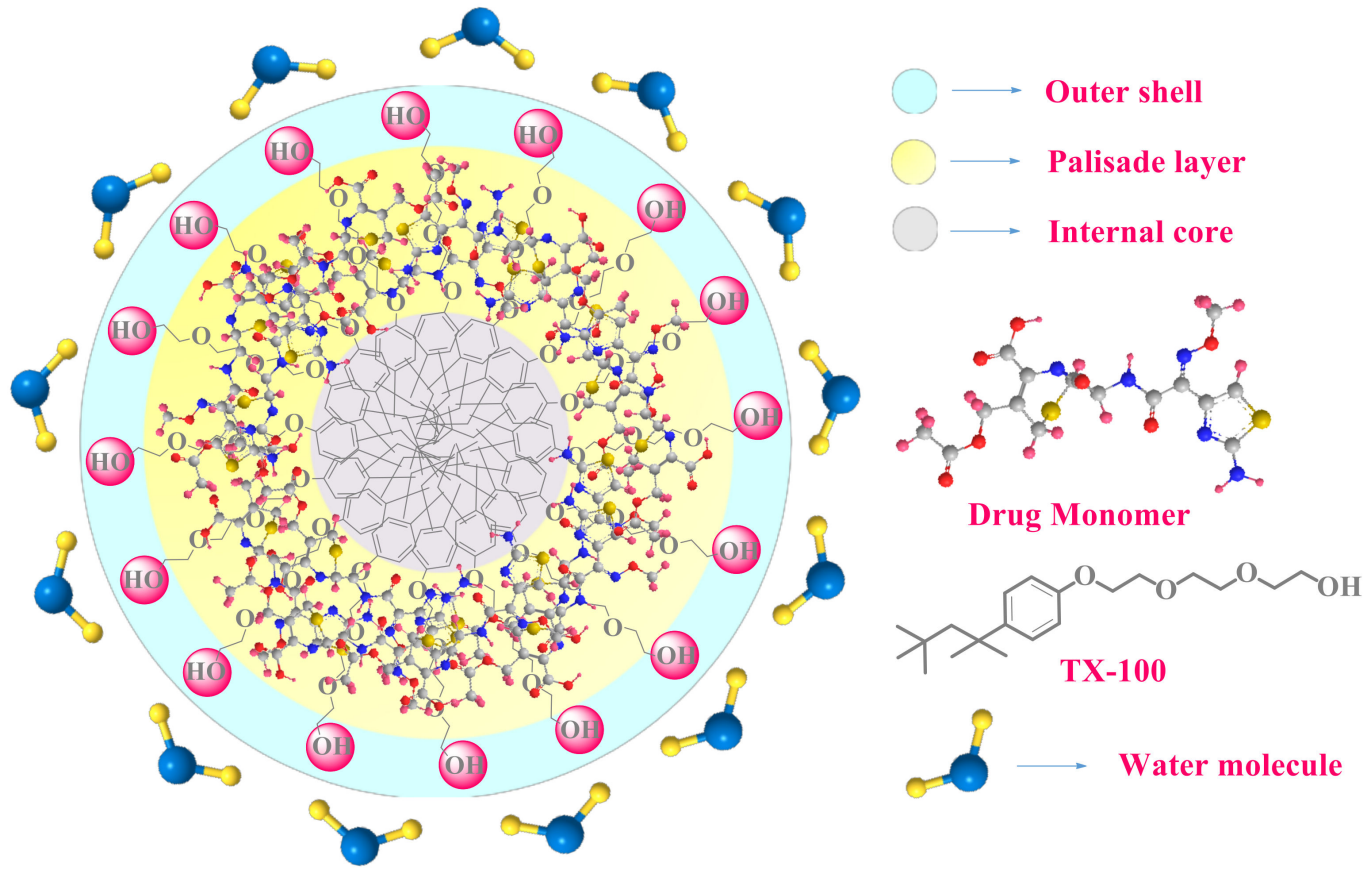
Locus of drug in the single micellar system of TX-100.

**Scheme 2:**
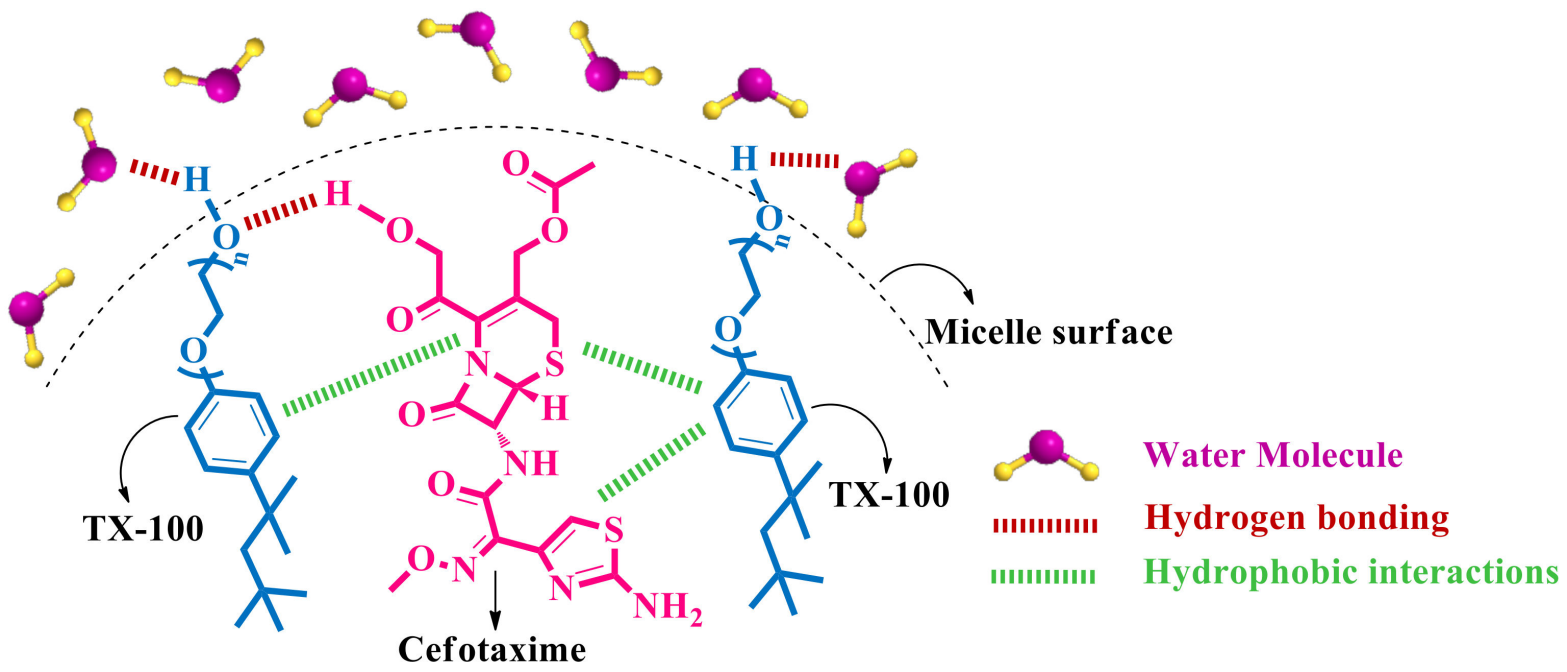
Insight into various possible interactions between drug monomer and the micellar system of TX-100.

Cefotaxime sodium (CEF), an antibiotic from the third generation of cephalosporin used to treat germs, is the medicine that was studied for its potential therapeutic action [56]. In order to improve the drug’s bioavailability, surfactants were used. To investigate surfactant drug interactions, UV-Visible spectroscopy was employed. Ionizable functional groups including carboxylic acid (-COOH) and amino groups (-NH_2_) are present in cefotaxime. Ethylene oxide (-OCH_2_CH_2_) units, which can form hydrogen bonds and interact electrostatically, are found in the structure of TX-100/TW-80. Electrostatic interactions made it easier for the antibiotics to bind with the micelles and maybe stabilize within [57]. Being nonionic surfactants, TX-100/TW-80 are also hydrophobic [58]. The side chains and fused rings are the main hydrophobic parts of the structure of cefotaxime. The hydrophobic core of nonionic micellar systems is made up of the hydrocarbon tails of molecules of surfactant, the polyoxyethylene casing (palisade layer), which surrounds the core, and the surface of the micelles. All of these are possible places for the drug to be located [59]. The hydrophobic portions of cefotaxime and the hydrophobic core of TX-100/TW-80 micelles may interact hydrophobically. Cefotaxime may become more soluble as a result of this interaction within the micelles hydrophobic interior, increasing its solubility in aqueous solutions. Scheme 1 and Scheme 2 depict where the drug monomer is located in the single micellar system of TX-100 and possible interactions between the drug monomer and surfactant molecules, respectively. From the calculations of the partitioning coefficient (K_x_), it is possible to estimate where drug molecules might be located in the micelles. Greater magnitudes of K_x_ in the case of TX-100 indicate accommodation of drug molecules close to the micelles palisade layer, and smaller values imply deep penetration [60].

**Scheme 3:**
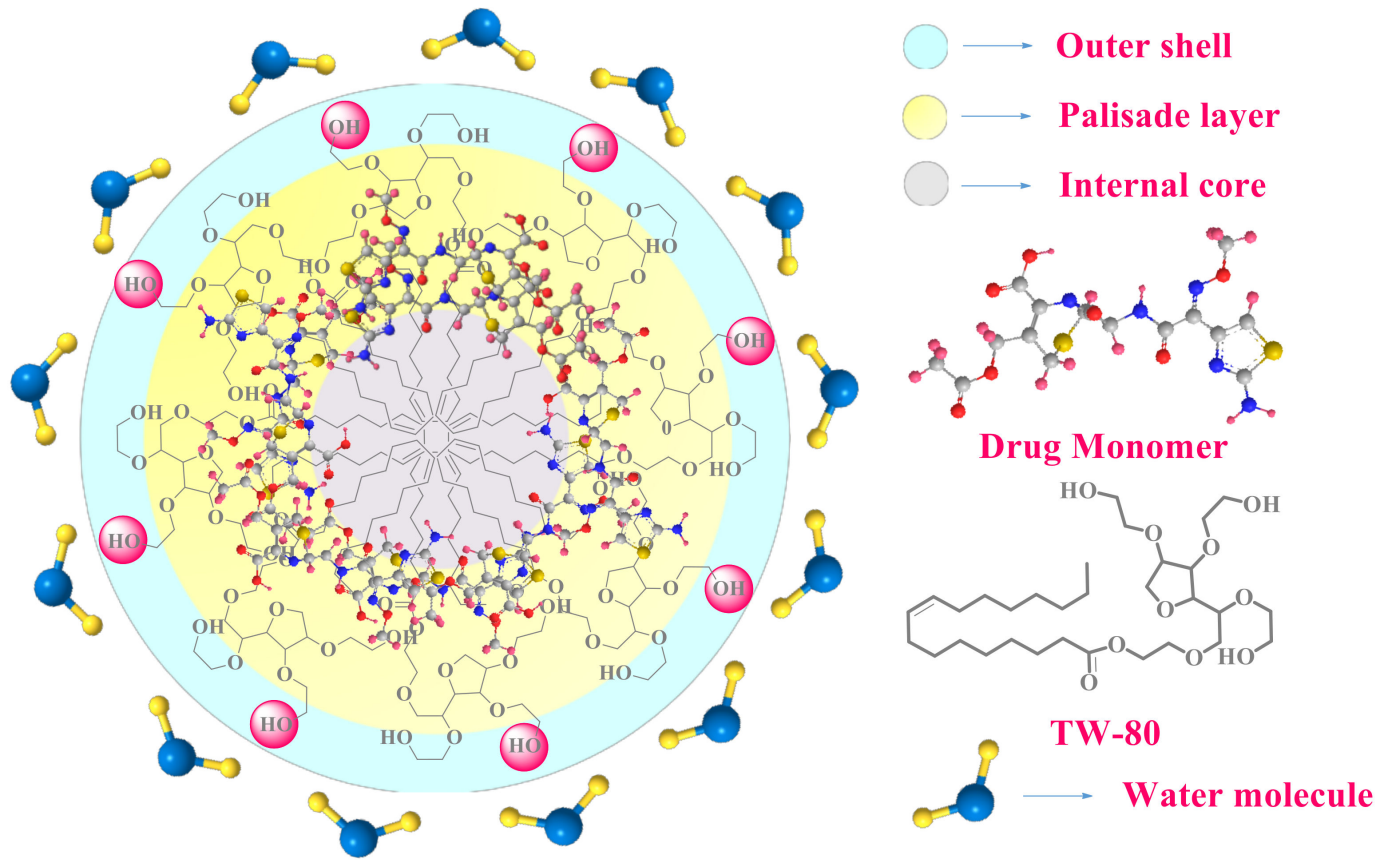
Locus of drug in the single micellar system of TW-80.

**Scheme 4:**
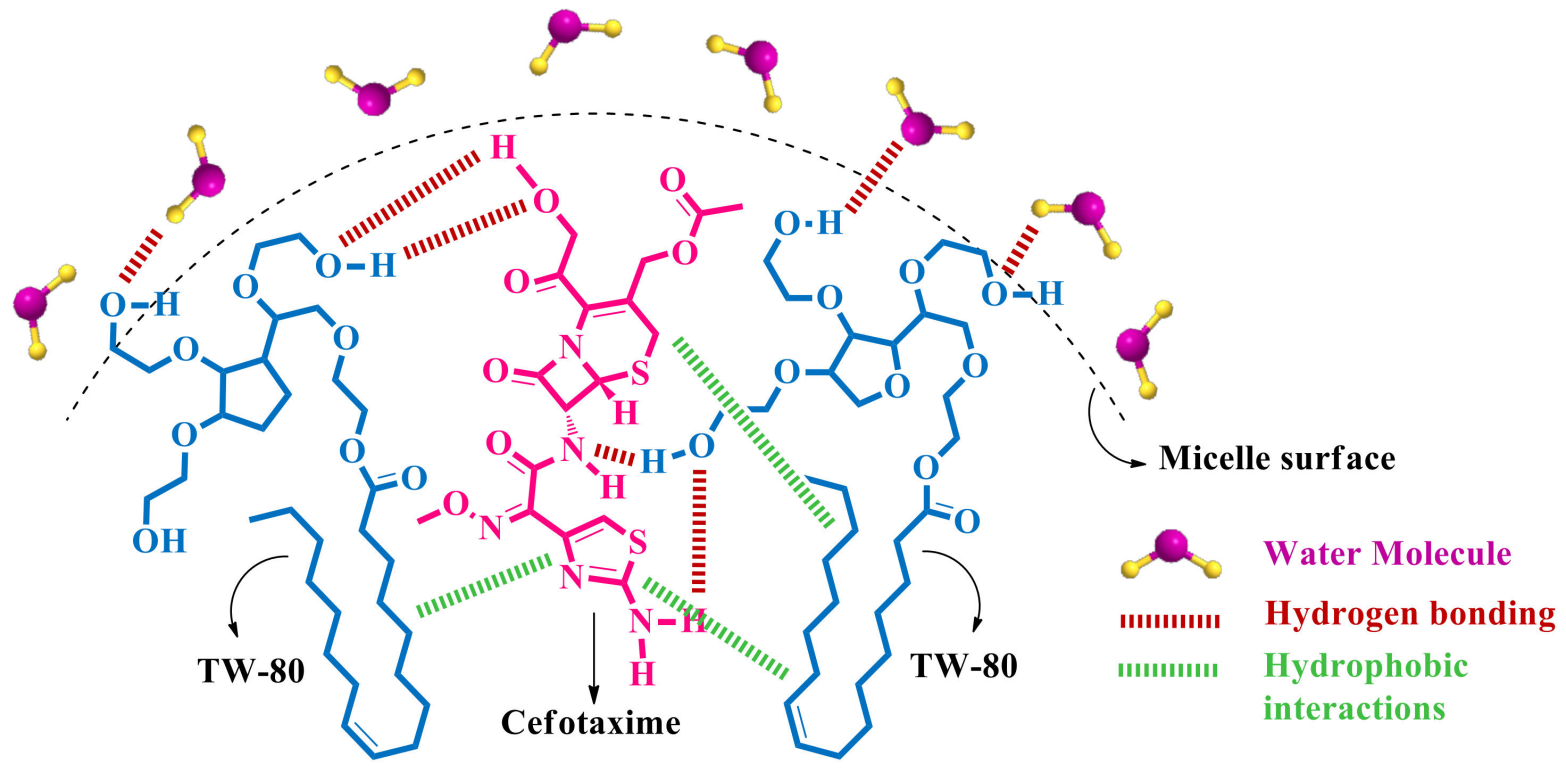
Insight into various possible interactions between drug monomer and the micellar system of TW-80.

In general, the efficacy of micelle solubilization increases with decreasing core polarity on a molar basis. The micellar core polarity for TX-100 is 1.40, and for TW-80 is 1.13, which indicates that, compared to TX-100, the micellar core of TW-80 offers a substantially more accepting environment for poorly water-soluble molecules in aqueous solution, indicating more effectiveness in solubilizing water-insoluble compounds [61]. The higher value of the binding coefficient for the micellar system of TW-80 compared to TX-100 also proves the deep penetration of drug molecules and thus demonstrates a higher solubilization power. Scheme 3 and Scheme 4 depict the position of the drug monomers in the single micellar system of TW-80 and possibilities for interaction between the monomer of the drug and surfactant molecules, respectively.

#### 2.3.2. Binding Nature of Cefotaxime in a Mixed Micellar System (Pre-Micellar TX-100 and Post-Micellar Tween 80)

**Scheme 5:**
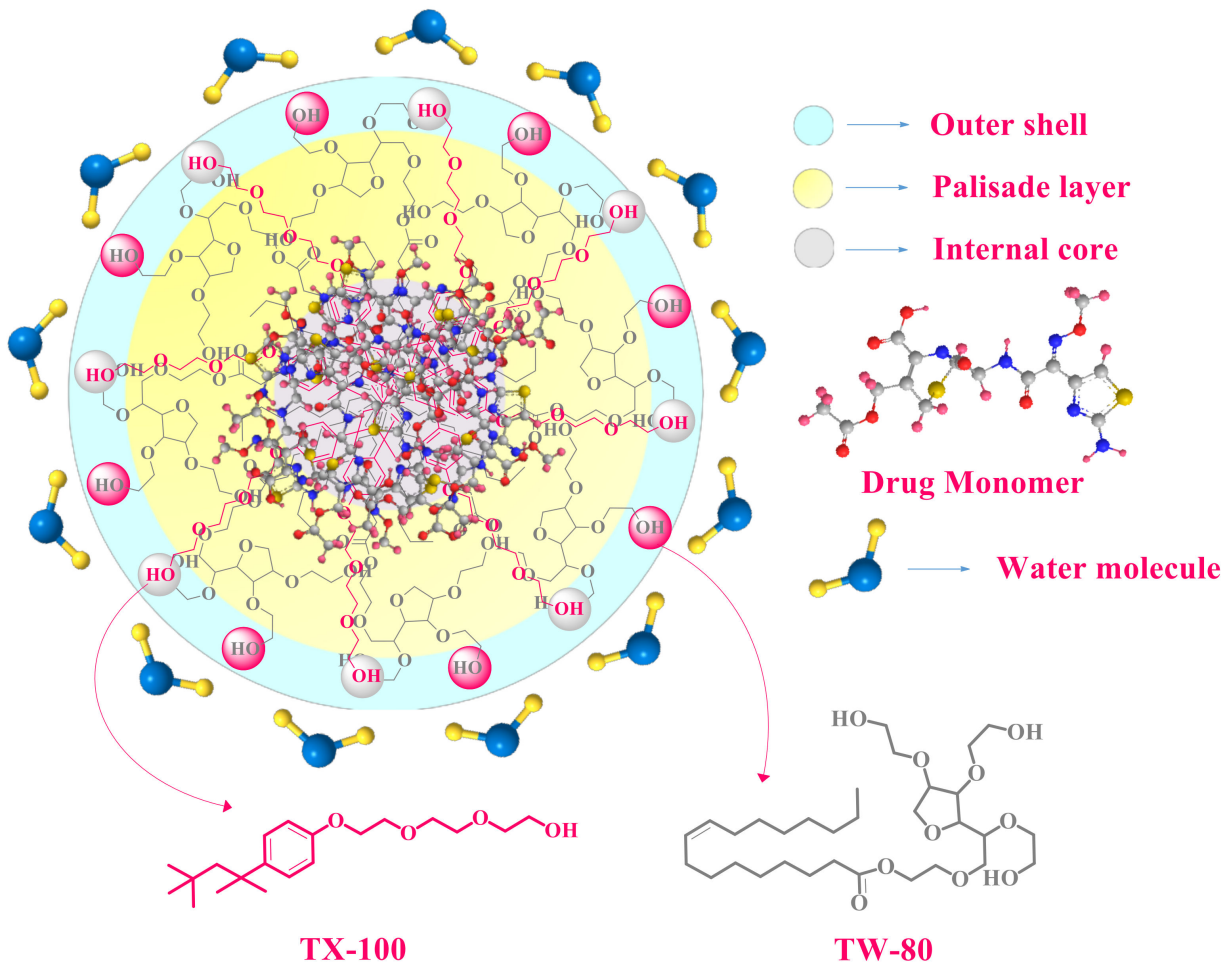
Locus of drug in the mixed micellar system of TX-100 and TW-80.

**Scheme 6:**
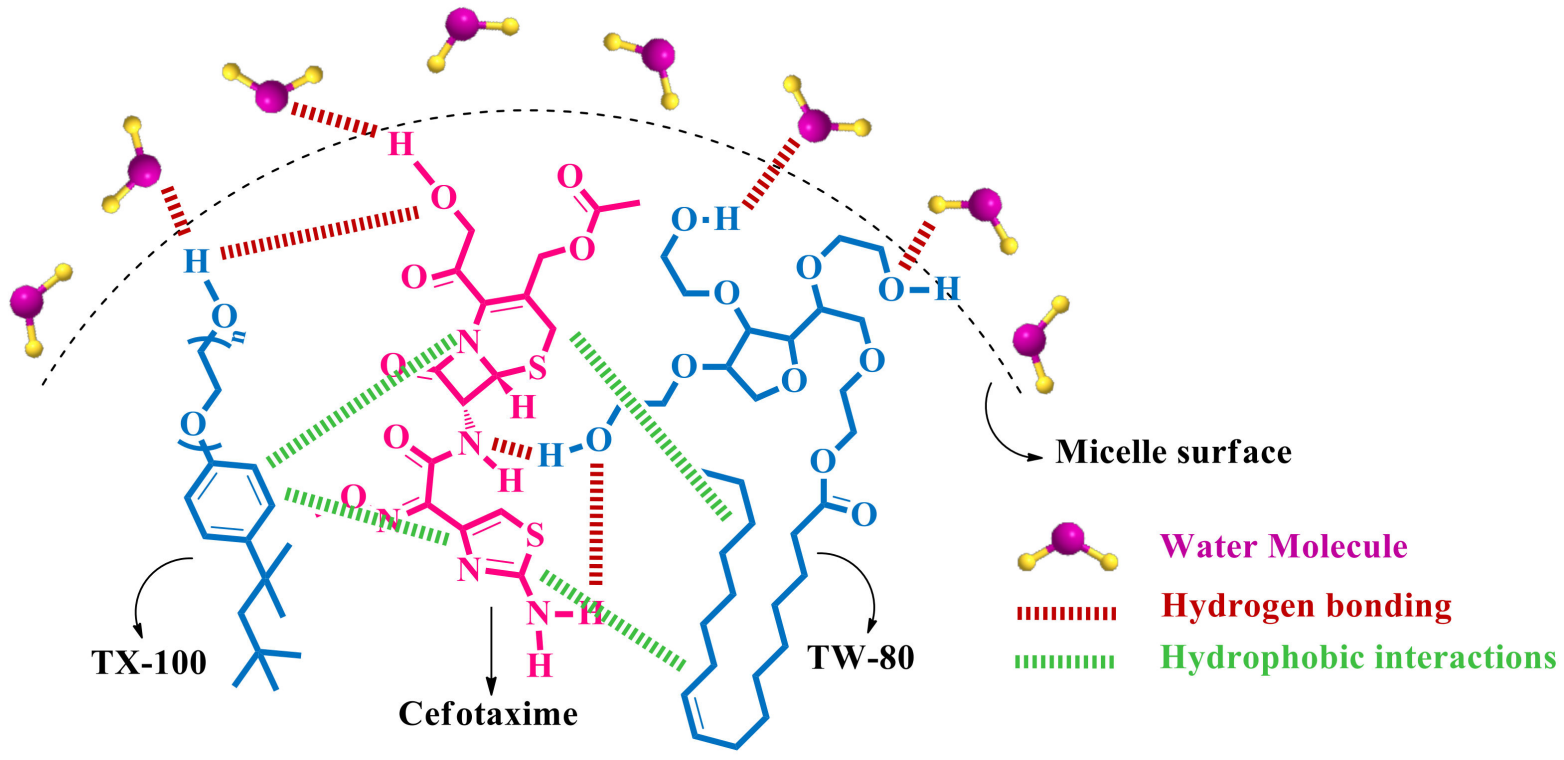
Insight into various possible interactions between drug monomer and mixed micellar system of TX-100 and TW-80.

In a mixed micellar solution that comprises both TX-100 and TW-80, the interaction process might be more complex. In the mixed micellar system, cefotaxime can interact with both the TX-100 and TW-80 micelles. It can interact with both the hydrophobic core of TX-100 micelles and the hydrophobic core of TW-80 micelles through hydrophobic interactions. This results in a combined solubilization activity from both surfactants result in deep penetration of the CEF inside the micelle as show in Scheme 5. The inclusion of two distinct types of micelles (TX-100 and TW-80) primarily distinguishes the mixed micellar system from the single micellar system. Cefotaxime can interact with both kinds of micelles in the mixed micellar system at the same time, taking advantage of the solubilization abilities of both surfactants as shown in Scheme 6. The application of a mixed micellar system shows a greater ability to raise the stability and solubility of cefotaxime in comparison to single micellar systems, potentially enhancing its therapeutic efficacy through changed physicochemical characteristics [42].

### 2.4. Study of Release Mechanism of Cefotaxime

The release of cefotaxime from the micelles of TX-100 and TW-80 in a biological system involves a combination of diffusion [62], environmental changes [63], and competition with biomolecules. Diffusion plays a significant role, with the high concentration of cefotaxime inside the micelle driving its movement towards areas of lower concentration in the surrounding biological fluid, such as blood or interstitial fluid [64]. Environmental alterations, such as temperature and pH, can influence micellar stability and structure. While TX-100 micelles are generally more pH-sensitive and relatively less stable over a wide pH range [65,66], TW-80 micelles are less pH-sensitive [67]. Because of the acidic environment in the stomach, the pH in the stomach is relatively low (typically around pH 1–3) [68]. At such low pH values, the acidic conditions can lead to the protonation of TX-100 surfactant molecules, which can disrupt the hydrophobic interactions within the micellar core [69]. The release of cefotaxime is facilitated by the micelle disintegration, making the drug available for absorption and therapeutic activity downstream from the gastrointestinal system. From the intestinal blood vessels, the absorbed cefotaxime is then transported to the liver to finally reach the systemic circulation [70].

As the ingested contents move from the stomach down to the small intestine, the value of pH gradually increases to a more neutral or slightly basic range (typically around pH 7–7.5) [71,72]. In this environment, the deprotonation process enhances the hydrophobic interactions within the micelles, restoring their structural integrity [59]. This increased stability can reduce the release of cefotaxime from the micellar core. TW-80 has a longer hydrophobic tail compared to TX-100, thus having stronger hydrophobic interactions between the tail groups, which contributes to greater micellar stability [73], making it less sensitive to pH changes. Changes in temperature can also affect micellar stability, with high temperatures causing disintegration and release, while low temperatures may enhance stability [74]. This effect is more prominent in temperature ranges approaching or exceeding the critical micelle temperature (CMT) for the surfactants, the temperature at which micelles begin formation or dissociation. Additionally, as micelles come into contact with the biological environment, various biomolecules, such as proteins and lipids, may have a higher affinity for the surfactant compared to cefotaxime due to their hydrophobic or hydrophilic regions. When these proteins and lipids interact with the surfactant molecules, they can displace or exchange with cefotaxime from the micellar core, resulting in the release of the encapsulated drug into surroundings [75].

## 3. Materials and Methods

### 3.1. Chemicals

All the materials that we utilized in this study, i.e., cefotaxime, surfactants (TX-100 and TW-80), solvent, were of analytical quality and used as collected, without additional purification. Water that had been double distilled was used for sample preparation and reactions. The stock solution of cefotaxime, as well as TX-100 and TW-80, was prepared in a molar concentration unit using distilled water. Cefotaxime was purchased from Sigma Aldrich (St. Louis, MO, USA). TX-100 and TW-80 were purchased from Daejung Chemicals and Metals CO. LTD., (Siheung-si, Republic of Korea). The mass fraction purity, CAS number, and molar mass of the compounds used are given in Table 5. The molecular structures of the used drug (CEF) and surfactants (TX-100 and TW-80) are depicted in Table 6.

### 3.2. Preparation of Stock Solutions and their Serial Dilution

The stock solution of the drug at the desired concentration of 5  ×  10^−5^ mol dm^−3^ was prepared for spectroscopic measurements. The ternary stock solutions of nonionic surfactants (water/drug/TX-100 and water/drug/TW-80) were prepared and diluted with the drug solution. Drug solubilization was investigated at various concentrations of TX-100 and TW-80. The TX-100 and TW-80 concentrations were set to range from 0.02 mM to 0.48 mM (0.02 mM increment) and from 0.0016 mM to 0.0384 mM (0.0016 mM increment), respectively. In the case of mixed micellization, the effect of TX-100 concentration on Tw-80 solubilization capacity was evaluated by introducing the pre-micellar amount of TX-100 to a micellar solution containing TW-80 for the purposes of avoiding the self-micellization of nonionic surfactants and maintaining the dominant character of TW-80 in the mixed micelles. For this, the quaternary solution of TX-100 was created by adding 0.0025, 0.0022, 0.0019, 0.0016, and 0.0013 mmol dm^−3^ of it to the existing ternary solution of TW-80. The solutions were kept for 24 h to allow for equilibrium before being used to take measurements [55,76].

### 3.3. Drug–Surfactant Interaction Study

The prepared drug solution was split into two portions. The pre-micellar to post-micellar concentration ranges of drug–surfactant solutions were prepared using one part as a reference solution and the other as a preparation step. The effect of various surfactant concentrations on the drug absorption spectra was then investigated in surfactant solutions with a constant drug concentration. Using a double-beam UV-Visible Spectrophotometer (C-7200S; Peak Instruments Inc., Houston, TX, USA), the UV-Visible absorption spectra of drugs were examined in the presence and absence of surfactants. Water was utilized as a reference for the measurement of basic absorption spectra while for the measurements of differential absorption, the drug solution was used as a reference. The ternary and quaternary solutions were added at various concentrations to the sample cell [19,32].

### 3.4. Calculated Parameters

#### 3.4.1. Partitioning Parameters

The ratio of the concentration of drug molecules in the micelle to that in the bulk aqueous solution is known as the partition coefficient. The UV-Visible absorption data were used to estimate the solubilization properties in terms of drug partition in the single and mixed surfactant systems. To investigate the distribution of drug molecules in solvent micellar systems, the Kawamura equation was applied [77], given as Equation (1):

(1) $$\frac{1}{\Delta A}=\frac{1}{K_{c}\Delta A_{\infty }{(C}_{d}+{\;C}_{s}^{m_{0}})}+\frac{1}{\Delta A_{\infty }}$$

where ΔA and ΔA_∞_ represent the differential absorbance values at normal and infinite concentrations, respectively. The precise concentration of the particular surfactant that is used is represented by $C_{s}^{m_{o}}$, whereas C_d_ is the drug concentration in mol/dm^3^. It is calculated as follows:

(2) $$C_{s}^{m_{o}}={\;C}_{s}-\mathrm{CM}C_{o}\;$$

where surfactant concentration is expressed as C_s_, and its CMC_o_, without addition of the drug, is expressed as CMC_o_. Using the aforementioned expression, the slope and intercept of the plot between the ΔA and (C_d_ + $C_{s}^{m_{0}}$) were used to calculate the value of the partition constant (K_c_). The coefficient of partition (K_x_) was subsequently determined using a value of K_c_ as follows:

(3) $$K_{c}={\;K}_{x}\times n_{w}$$

where n_w_ stands for the number of moles of water/dm^3^. The following formulas were then applied to K_x_ values to determine how the free energies of these processes changed:

(4) $$\Delta G_{p}=-\mathrm{RT}\ln K_{x}\;$$

#### 3.4.2. Binding Parameters

The Benesi-Hildebrand equation was used for calculating the degree of binding within the drug-TX-100/TW-80 system [78]:

(5) $$\frac{C_{s}C_{d}}{\Delta A}=\frac{C_{s}}{\Delta \varepsilon l}+\frac{1}{K_{b}\Delta \varepsilon l}\;$$

where C_d_ stands for the drug concentration, and C_s_ for the surfactant concentration. Additionally, *l* is the path length, Δε is the change in absorption coefficient, ΔA stands for differential absorbance, and K_b_ is the binding constant. The following relation was used to compute the value of the standard free energy change in binding:

(6) $$\Delta G_{b}=-\mathrm{RT}\ln K_{b}\;$$

## 4. Conclusions

This study reports the interactional study of cefotaxime in the presence of nonionic surfactants, viz., Triton X-100 and Tween 80. All the investigations were effectively conducted using UV-Visible spectroscopic measurements to study the interactions of drugs with nonionic surfactants and the importance of surfactants in the solubilization of the drugs in this work. Using the spectroscopic data, the partition constant (K_c_), partition coefficient (K_x_), binding constant (K_b_), Gibbs free energy of partition (ΔG_p_), and Gibbs free energy of binding (ΔG_b_) were determined. Based on these findings, we concluded that while single surfactant systems do improve hydrophobic drug solubility in water, they are not very stable and have a negligible drug-storing capacity. These issues were fixed by combining TX-100, Tween-80, and nonionic surfactants to create a mixed micellar system. The enhancement of solubilization of cefotaxime in the mixed micellar system (TW-80 + TX-100) was observed in contrast to single micellar media of both TW-80 and TX-100. The addition of TX-100 enhanced the hydrophobicity of the micellar media. Mixing nonionic surfactants resulted in better performance in drug entrapment. The mixing process proved to achieve a higher degree of solubilization for the drug. The addition of TX-100 enhanced the hydrophobicity of the micellar media. The results of these experiments revealed that a mixed micellar media of TW-80 and TX-100 is the best medium for entrapment of drugs. The negative values for ΔG_b_ and ΔG_p_ indicate its spontaneity. Our results imply that using the designated surfactant is a great method for enhancing cefotaxime solubility and contributes significantly to addressing the solubility problems of drugs, which pose a challenging task for the pharmaceutical industry. While micellar systems revealed significant advantages in terms of drug solubility and potential targeted administration in our research, we acknowledge that these systems have certain limits. Our research reveals that micellar systems are sensitive to environmental conditions such as pH and temperature, potentially affecting their stability and drug release kinetics. Despite the effective solubilization of our drug, we observed limitations in drug-loading capacity at higher concentrations of TX-100 in the mixed micellar system of TW-80 and TX-100, which could affect the delivery of larger drug doses.

## Figures and Tables

**Table 1:** Calculations for the partitioning coefficient and binding coefficient of CEF in TX-100 micellar media.

C_s_ (mM)	∆A	1/∆A	C_d_ (mM)	CMC_o_ (mM)	C_s_ − CMC_o_ (mol dm^−3^)	$C_{d}\;+\;C_{s}^{m_{o}}$ (mol dm^−3^)	$1/(C_{d}\;+\;C_{s}^{m_{o}}$) (mol^−1^ dm^3^)	C_s_C_d_/∆A × 10^−6^ (mol^−2^ dm^6^)
0.22	1.448	0.691	0.05	0.2	0.00002	0.00007	14286	0.0076
0.24	1.454	0.688	0.05	0.2	0.00004	0.00009	11111	0.0083
0.26	1.458	0.686	0.05	0.2	0.00006	0.00011	9091	0.0089
0.28	1.460	0.685	0.05	0.2	0.00008	0.00013	7692	0.0096
0.30	1.464	0.683	0.05	0.2	0.00010	0.00015	6667	0.0102
0.32	1.469	0.681	0.05	0.2	0.00012	0.00017	5882	0.0109

**Table 2:** Calculations for the partitioning coefficient and binding coefficient of CEF in TW-80 micellar media.

C_s_ (mM)	∆A	1/∆A	C_d_ (mM)	CMC_o_ (mM)	C_s_ − CMC_o_ (mol dm^−3^)	$C_{d}\;+\;C_{s}^{m_{o}}$ (mol dm^−3^)	$1/(C_{d}\;+\;C_{s}^{m_{o}}$) (mol^−1^ dm^3^)	C_s_C_d_/∆A × 10^−6^ (mol^−2^ dm^6^)
0.016	0.925	1.081	0.05	0.015	0.0000010	0.000051	19608	0.00086
0.018	0.933	1.072	0.05	0.015	0.0000026	0.000053	19011	0.00094
0.019	0.945	1.058	0.05	0.015	0.0000042	0.000054	18450	0.00102
0.021	0.949	1.054	0.05	0.015	0.0000058	0.000056	17921	0.00110
0.022	0.957	1.045	0.05	0.015	0.0000074	0.000057	17422	0.00117
0.024	0.961	1.041	0.05	0.015	0.0000090	0.000059	16949	0.00125

**Table 3:** Partition and binding coefficients of CEF in a single micellar system.

System	K_x_ × 10^6^	$\Delta G_{p}$ (kJ/mol)	K_b_ × 10^5^ (dm^3^/mol)	$\Delta G_{b}$ (kJ/mol)
CEF/TX-100	33.78	−43.46	0.98	−28.47
CEF/Tw-80	2.78	−37.20	4.83	−32.43

**Table 4:** Partition and binding parameters of the CEF in mixed micellar media.

System	K_x_ × 10^6^	$\Delta G_{p}$ (kJ/mol)	K_b_ × 10^5^ (dm^3^/mol)	$\Delta G_{b}$ (kJ/mol)
0.0013	2.46	−36.89	2.54	−30.83
0.0016	4.21	−38.24	4.08	−32.01
0.0019	5.48	−38.90	4.86	−32.44
0.0022	3.88	−38.04	4.07	−32.00
0.0025	3.23	−37.58	3.26	−31.46

**Table 5:** Chemicals used in this work and their specifications.

Chemicals	Sources	Mass Fraction Purity	CAS Number	Molecular Mass
Cefotaxime	Sigma Aldrich	>98% (HPLC)	104376-79-6	455.47 gmol^−1^
TX-100	Daejung Chemicals and Metals Co., Ltd.	98% purity	9002-93-1	647.0 gmol^−1^
Tween-80	Daejung Chemicals and Metals Co., Ltd.	98.9%	9005-65-6	1310 gmol^−1^

**Table 6:** Molecular illustration of surfactants and reactive drugs.

Molecules	Abbreviations	Structures
Cefotaxime	CEF	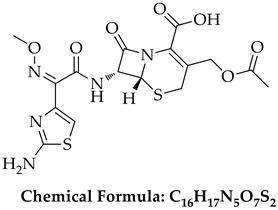
Triton X-100	TX-100	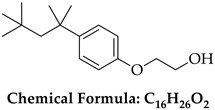
Tween-80	TW-80	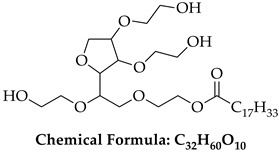

## Data Availability

The data presented in this study are available on request from the corresponding author.

## References

[1] Zhan X., Wu Z., Chen Z., Cui X. (2022). Mechanism of the Micellar Solubilization of Curcumin by Mixed Surfactants of SDS and Brij35 via NMR Spectroscopy. Molecules.

[2] Clulow A.J., Barber B., Salim M., Ryan T., Boyd B.J. (2020). Synergistic and antagonistic effects of non-ionic surfactants with bile salt+ phospholipid mixed micelles on the solubility of poorly water-soluble drugs. Int. J. Pharm..

[3] Mladěnka P., Applová L., Patočka J., Costa V.M., Remiao F., Pourová J., Mladěnka A., Karlíčková J., Jahodář L., Vopršalová M. (2018). Comprehensive review of cardiovascular toxicity of drugs and related agents. Med. Res. Rev..

[4] Srivastava A., Uchiyama H., Wada Y., Hatanaka Y., Shirakawa Y., Kadota K., Tozuka Y. (2019). Mixed micelles of the antihistaminic cationic drug diphenhydramine hydrochloride with anionic and non-ionic surfactants show improved solubility, drug release and cytotoxicity of ethenzamide. J. Mol. Liq..

[5] Seleci D.A., Seleci M., Walter J.-G., Stahl F., Scheper T. (2016). Niosomes as nanoparticular drug carriers: Fundamentals and recent applications. J. Nanomater..

[6] Aguirre-Ramírez M., Silva-Jiménez H., Banat I.M., Díaz De Rienzo M. (2021). Surfactants: Physicochemical interactions with biological macromolecules. Biotechnol. Lett..

[7] Yusaf A., Usman M., Mansha A., Saeed M., Ahmad M., Siddiq M. (2022). Micellar-enhanced ultrafiltration (MEUF) for removal of rhodamine B (RhB) from aqueous system. J. Dispers. Sci. Technol..

[8] Yusaf A., Usman M., Mansha A., Siddiq M., Amjad Z., Irshad S., Sultana H. (2022). Self-organised surfactant assemblies as nanostructured dye carriers: A mixed micellar comparative approach for enhanced dye solubilisation. Int. J. Environ. Anal. Chem..

[9] Shakeel F., Alshehri S., Ibrahim M.A., Altamimi M., Haq N., Elzayat E.M., Shazly G.A. (2021). Solubilization and thermodynamic properties of simvastatin in various micellar solutions of different non-ionic surfactants: Computational modeling and solubilization capacity. PLoS ONE.

[10] Yusaf A., Usman M., Ahmad M., Siddiq M., Mansha A., Al-Hussain S.A., Zaki M.E., Rehman H.F. (2022). Highly Selective Methodology for Entrapment and Subsequent Removal of Cobalt (II) Ions under Optimized Conditions by Micellar-Enhanced Ultrafiltration. Molecules.

[11] Sharma N., Madan P., Lin S. (2016). Effect of process and formulation variables on the preparation of parenteral paclitaxel-loaded biodegradable polymeric nanoparticles: A co-surfactant study. Asian J. Pharm. Sci..

[12] Nishikido N. (2020). Solubilization in mixed micelles. Solubilization in Surfactant Aggregates.

[13] Srivastava A., Kumar M., Deb D.K., Muzaffar F., Singh S. (2022). Utilization of amphiphilic antihistamines drugs to enhance micellization of anionic surfactant and improve the binding and solubility of Itraconazole drug. J. Mol. Liq..

[14] Silva A.C., Lopes C.M., MS Lobo J., Amaral M.H. (2015). Delivery systems for biopharmaceuticals. Part II: Liposomes, micelles, microemulsions and dendrimers. Curr. Pharm. Biotechnol..

[15] Alavi M., Karimi N., Safaei M. (2017). Application of various types of liposomes in drug delivery systems. Adv. Pharm. Bull..

[16] Wakaskar R.R. (2018). General overview of lipid–polymer hybrid nanoparticles, dendrimers, micelles, liposomes, spongosomes and cubosomes. J. Drug Target..

[17] Baer B., Souza L.M.P., Pimentel A.S., Veldhuizen R.A. (2019). New insights into exogenous surfactant as a carrier of pulmonary therapeutics. Biochem. Pharmacol..

[18] Hwang D., Ramsey J.D., Kabanov A.V. (2020). Polymeric micelles for the delivery of poorly soluble drugs: From nanoformulation to clinical approval. Adv. Drug Deliv. Rev..

[19] Akhter K., Ullah K., Talat R., Haider A., Khalid N., Ullah F., Ali S. (2019). Synthesis and characterization of cationic surfactants and their interactions with drug and metal complexes. Heliyon.

[20] Lalthlengliani J., Gurung J., Pulikkal A.K. (2022). Solubilization of aqueous-insoluble phenothiazine drug in TX-100 micellar solution and interactions of cationic/anionic surfactants with phenothiazine–TX-100 system. J. Mol. Liq..

[21] Alizadeh M.N., Shayanfar A., Jouyban A. (2018). Solubilization of drugs using sodium lauryl sulfate: Experimental data and modeling. J. Mol. Liq..

[22] Muhammad M.T., Khan M.N. (2021). Spectrophotometric investigation of surfactants mediated aqueous solubilization of Nile blue. J. Mol. Liq..

[23] Ullah F., Ullah S., Khan M.F.A., Khan F., Tareen N.H.K. (2022). Enhancement in aqueous solubility of sulindac medicine by using the micellar solution of ionic and non-ionic surfactants. Biomed. Lett..

[24] Majumder N., Das N.G., Das S.K. (2020). Polymeric micelles for anticancer drug delivery. Ther. Deliv..

[25] Azum N., Rub M.A., Asiri A.M., Bawazeer W.A. (2017). Micellar and interfacial properties of amphiphilic drug–non-ionic surfactants mixed systems: Surface tension, fluorescence and UV–vis studies. Colloids Surf. A Physicochem. Eng. Asp..

[26] Lu X., Li M., Arce F.A., Ling J., Setiawan N., Wang Y., Shi X., Campbell H.R., Nethercott M.J., Xu W. (2021). Mechanistic Investigation of Drug Supersaturation in the Presence of Polysorbates as Solubilizing Additives by Solution Nuclear Magnetic Resonance Spectroscopy. Mol. Pharm..

[27] Maity B., Chatterjee A., Ahmed S.A., Seth D. (2015). Interaction of the nonsteroidal anti-inflammatory drug indomethacin with micelles and its release. J. Phys. Chem. B.

[28] Talele P., Choudhary S., Kishore N. (2016). Understanding thermodynamics of drug partitioning in micelles and delivery to proteins: Studies with naproxen, diclofenac sodium, tetradecyltrimethylammonium bromide, and bovine serum albumin. J. Chem. Thermodyn..

[29] Sar P., Ghosh A., Scarso A., Saha B. (2019). Surfactant for better tomorrow: Applied aspect of surfactant aggregates from laboratory to industry. Res. Chem. Intermed..

[30] Bandivadekar M., Pancholi S., Kaul-Ghanekar R., Choudhari A., Koppikar S. (2013). Single non-ionic surfactant based self-nanoemulsifying drug delivery systems: Formulation, characterization, cytotoxicity and permeability enhancement study. Drug Dev. Ind. Pharm..

[31] Teleb M.S., Mohammed S.F., Gaballa A.S. (2021). Syntheses and identi ication of cefotaxime-non-transition metal complexes. Int. J. Res. Pharm. Sci..

[32] Irshad S., Sultana H., Usman M., Saeed M., Akram N., Yusaf A., Rehman A. (2021). Solubilization of direct dyes in single and mixed surfactant system: A comparative study. J. Mol. Liq..

[33] Noor S., Taj M.B. (2021). Mixed-micellar approach for enhanced dye entrapment: A spectroscopic study. J. Mol. Liq..

[34] Brandis A., Mazor O., Neumark E., Rosenbach-Belkin V., Salomon Y., Scherz A. (2005). Novel Water-soluble Bacteriochlorophyll Derivatives for Vascular-targeted Photodynamic Therapy: Synthesis, Solubility, Phototoxicity and the Effect of Serum Proteins. Photochem. Photobiol..

[35] Rehman A., Nisa M.U., Usman M., Ahmad Z., Bokhari T.H., Rahman H.M.A.U., Rasheed A., Kiran L. (2021). Application of cationic-nonionic surfactant based nanostructured dye carriers: Mixed micellar solubilization. J. Mol. Liq..

[36] Zhang L., Chai X., Sun P., Yuan B., Jiang B., Zhang X., Liu M. (2019). The study of the aggregated pattern of TX100 micelle by using solvent paramagnetic relaxation enhancements. Molecules.

[37] Anand U., Jash C., Mukherjee S. (2011). Spectroscopic determination of Critical Micelle Concentration in aqueous and non-aqueous media using a non-invasive method. J. Colloid Interface Sci..

[38] Mahmood M.E., Al-Koofee D.A. (2013). Effect of temperature changes on critical micelle concentration for tween series surfactant. Glob. J. Sci. Front. Res. Chem..

[39] Hanif S., Usman M., Hussain A., Rasool N., Zubair M., Rana U.A. (2015). Solubilization of Benzothiazole (BNZ) by micellar media of Sodium dodecyl sulphate and Cetyl trimethylammonium bromide. J. Mol. Liq..

[40] Usman M., Rashid M.A., Mansha A., Siddiq M. (2013). Thermodynamic solution properties of pefloxacin mesylate and its interactions with organized assemblies of anionic surfactant, sodium dodecyl sulphate. Thermochim. Acta.

[41] Nazar M.F., Shah S.S., Khosa M.A. (2010). Interaction of azo dye with cationic surfactant under different pH conditions. J. Surfactants Deterg..

[42] Yusaf A., Usman M., Siddiq M., Bakhtiar M., Mansha A., Shaukat S., Rehman H.F. (2022). Mixed Micellar Solubilization of Naphthol Green B Followed by Its Removal from Synthetic Effluent by Micellar-Enhanced Ultrafiltration under Optimized Conditions. Molecules.

[43] Acosta E., Bisceglia M., Kurlat D. (2005). Self-aggregation in aqueous TRITON X-100 solutionsnear CMC. Phys. Chem. Liq..

[44] Stubičar N., Petres J. (1981). Micelle formation by tritons in aqueous solutions. Croat. Chem. Acta.

[45] Serafini P., Leyes M.F., Pereyra R.B., Schulz E.P., Durand G.A., Schulz P.C., Ritacco H.A. (2018). The aqueous Triton X-100–dodecyltrimethylammonium bromidemicellar mixed system. Experimental results and thermodynamic analysis. Colloids Surf. A Physicochem. Eng. Asp..

[46] Amani A., York P., de Waard H., Anwar J. (2011). Molecular dynamics simulation of a polysorbate 80 micelle in water. Soft Matter.

[47] Berezin D.B., Kustov A.V., Krest’yaninov M.A., Shukhto O.V., Batov D.V., Natal’ya V.K. (2019). The behavior of monocationic chlorin in water and aqueous solutions of non-ionic surfactant Tween 80 and potassium iodide. J. Mol. Liq..

[48] Karanth S., Iyyaswami R. (2021). Analysis of ionic and nonionic surfactants blends used for the reverse micellar extraction of Lactoperoxidase from whey. Asia-Pac. J. Chem. Eng..

[49] Kronberg B. (2016). The hydrophobic effect. Curr. Opin. Colloid Interface Sci..

[50] Reeve J.R., Thomas R.K., Penfold J. (2021). Surface activity of ethoxylate surfactants with different hydrophobic architectures: The effect of layer substructure on surface tension and adsorption. Langmuir.

[51] Shaban S.M., Abd Elsamad S., Tawfik S.M., Adel A.-H., Aiad I. (2020). Studying surface and thermodynamic behavior of a new multi-hydroxyl Gemini cationic surfactant and investigating their performance as corrosion inhibitor and biocide. J. Mol. Liq..

[52] Rub M.A., Azum N., Kumar D., Asiri A.M., Marwani H.M. (2014). Micellization and microstructural studies between amphiphilic drug ibuprofen with non-ionic surfactant in aqueous urea solution. J. Chem. Thermodyn..

[53] Maswal M., Chat O.A., Jabeen S., Ashraf U., Masrat R., Shah R.A., Dar A.A. (2015). Solubilization and co-solubilization of carbamazepine and nifedipine in mixed micellar systems: Insights from surface tension, electronic absorption, fluorescence and HPLC measurements. RSC Adv..

[54] Bhat P.A., Rather G.M., Dar A.A. (2009). Effect of surfactant mixing on partitioning of model hydrophobic drug, naproxen, between aqueous and micellar phases. J. Phys. Chem. B.

[55] Irshad S., Sultana H., Usman M., Akram N., Farooqi Z.H., Yusaf A., Nazar M.F. (2021). Solubilization of direct black 2 in mixed micellar media: Insights from spectroscopic and conductometric measurements. J. Dispers. Sci. Technol..

[56] Al Hagbani T., Rizvi S.M.D., Hussain T., Mehmood K., Rafi Z., Moin A., Abu Lila A.S., Alshammari F., Khafagy E.-S., Rahamathulla M. (2022). Cefotaxime mediated synthesis of gold nanoparticles: Characterization and antibacterial activity. Polymers.

[57] Hoque M.A., Mahbub S., Hossain M.D., Khan M.A., Khan J.M., Malik A., Ahmed A., Ahmed M.Z. (2021). Influence of NaCl and temperature on the interaction between cephradine monohydrate and surfactants: Conductivity and UV–visible measurements. J. Mol. Liq..

[58] Ishkhanyan H., Rhys N.H., Barlow D.J., Lawrence M.J., Lorenz C.D. (2022). Impact of drug aggregation on the structural and dynamic properties of Triton X-100 micelles. Nanoscale.

[59] Enache M., Volanschi E. (2012). Spectroscopic investigations of the molecular interaction of anticancer drug mitoxantrone with non-ionic surfactant micelles. J. Pharm. Pharmacol..

[60] Rosen M.J., Kunjappu J.T. (2012). Surfactants and Interfacial Phenomena.

[61] Jiao J. (2008). Polyoxyethylated nonionic surfactants and their applications in topical ocular drug delivery. Adv. Drug Deliv. Rev..

[62] Pavlović N., Goločorbin-Kon S., Ðanić M., Stanimirov B., Al-Salami H., Stankov K., Mikov M. (2018). Bile acids and their derivatives as potential modifiers of drug release and pharmacokinetic profiles. Front. Pharmacol..

[63] Islam M.N., Rub M.A., Islam M.R., Goni M.A., Rana S., Kumar D., Asiri A.M., Alghamdi Y.G., Hoque M.A., Kabir S.E. (2023). Physico-chemical study of the effects of electrolytes and hydrotropes on the clouding development of TX-100 and ceftriaxone sodium drug mixture. J. Mol. Liq..

[64] Ghezzi M., Pescina S., Padula C., Santi P., Del Favero E., Cantù L., Nicoli S. (2021). Polymeric micelles in drug delivery: An insight of the techniques for their characterization and assessment in biorelevant conditions. J. Control. Release.

[65] Patel V., Bharatiya B., Ray D., Aswal V., Bahadur P. (2015). Investigations on microstructural changes in pH responsive mixed micelles of Triton X-100 and bile salt. J. Colloid Interface Sci..

[66] Leroux J.-C., Roux E., Le Garrec D., Hong K., Drummond D.C. (2001). N-isopropylacrylamide copolymers for the preparation of pH-sensitive liposomes and polymeric micelles. J. Control. Release.

[67] Prabhu A.A.M., Rajendiran N., Sathiyaseelan K. (2021). Equilibrium Constant, Concentrations Study of p-Aminobenzoic Acid in Anionic, Cationic and Non-ionic Surfactants. Science.

[68] Beasley D.E., Koltz A.M., Lambert J.E., Fierer N., Dunn R.R. (2015). The evolution of stomach acidity and its relevance to the human microbiome. PLoS ONE.

[69] Asman S., Abas N.A. (2018). Triton X-100/β-Cyclodextrin Cloud Point Extraction for Removal of Phenol Using Different of Sodium Salts as Inducing Phase Separation Agent. Asian J. Chem..

[70] Zhang Z., Lu Y., Qi J., Wu W. (2021). An update on oral drug delivery via intestinal lymphatic transport. Acta Pharm. Sin. B.

[71] Abuhelwa A.Y., Williams D.B., Upton R.N., Foster D.J. (2017). Food, gastrointestinal pH, and models of oral drug absorption. Eur. J. Pharm. Biopharm..

[72] Hens B., Tsume Y., Bermejo M., Paixao P., Koenigsknecht M.J., Baker J.R., Hasler W.L., Lionberger R., Fan J., Dickens J. (2017). Low buffer capacity and alternating motility along the human gastrointestinal tract: Implications for in vivo dissolution and absorption of ionizable drugs. Mol. Pharm..

[73] Sun Y., Li M., Gu X., Danish M., Shan A., Ali M., Qiu Z., Sui Q., Lyu S. (2021). Mechanism of surfactant in trichloroethene degradation in aqueous solution by sodium persulfate activated with chelated-Fe (II). J. Hazard. Mater..

[74] Ozkan A.D., Tekinay A.B., Guler M.O., Tekin E.D. (2016). Effects of temperature, pH and counterions on the stability of peptide amphiphile nanofiber structures. RSC Adv..

[75] Guagliardo R., Perez-Gil J., De Smedt S., Raemdonck K. (2018). Pulmonary surfactant and drug delivery: Focusing on the role of surfactant proteins. J. Control. Release.

[76] Noor S., Taj M.B., Naz I. (2022). Comparative solubilization of reactive dyes in single and mixed surfactants. J. Dispers. Sci. Technol..

[77] Yusaf A., Usman M., Ibrahim M., Mansha A., ul Haq A., Rehman H.F., Ali M. (2023). Mixed micellar solubilization for procion blue MxR entrapment and optimization of necessary parameters for micellar enhanced ultrafiltration. Chemosphere.

[78] Noor S., Taj M.B., Ashar A. (2021). Solubilization of cationic dye in single and mixed micellar media. J. Mol. Liq..

